# The Spinal Cord Stimulation Trial Success Score (STSS): A Narrative Review and Evidence-Informed Conceptual Framework for Structured Candidate Assessment

**DOI:** 10.3390/jcm15134849

**Published:** 2026-06-23

**Authors:** Jakub Wiśniewski, Mateusz Szczupak, Paweł Jan Winklewski, Anna Barbara Marcinkowska

**Affiliations:** 1 Department of Neurosurgery, Nicolaus Copernicus Hospital, 80-803 Gdańsk, Poland; 2 Department of Anaesthesiology and Intensive Therapy, Nicolaus Copernicus Hospital, 80-803 Gdansk, Poland; szczupak.mateusz@icloud.com; 3 Department of Neurophysiology, Neuropsychology and Neuroinformatics, Medical University of Gdansk, 80-210 Gdansk, Poland; pawelwinklewski@wp.pl; 4 2nd Department of Radiology, Medical University of Gdansk, 80-210 Gdansk, Poland

**Keywords:** spinal cord stimulation, patient selection, neuromodulation, chronic pain, prognostic domains, clinical framework, trial stimulation, shared decision-making

## Abstract

**Background:** Spinal cord stimulation (SCS) is an established intervention for refractory chronic neuropathic pain, but response to trial stimulation and long-term benefit remain heterogeneous. Clinicians need practical tools to document patient-selection domains discussed in the neuromodulation literature without overstating the precision of currently available evidence. **Methods:** We conducted a narrative synthesis of randomized trials, cohort studies, registry analyses, systematic reviews, and consensus recommendations addressing SCS outcomes and candidate selection. The objective was not to derive or validate a multivariable prediction model, but to construct a transparent, bedside-oriented framework organizing clinically accessible domains relevant to SCS trial candidacy. **Results:** Six domains were incorporated into the proposed SCS Trial Success Score (STSS): primary indication, psychological status, smoking status, opioid burden, body mass index, and pain duration. The resulting 0 to 12 point score is presented as an evidence-informed clinical profile rather than a validated prognostic instrument. Three descriptive categories are proposed: more favorable profile, optimization-sensitive profile, and less favorable profile. These categories are intended to guide documentation, shared decision-making, and optimization of modifiable factors, not to determine eligibility automatically. **Conclusions:** Pending prospective validation, checklist-mode use is the preferred interim application of the STSS. The framework may support structured pre-trial assessment, identification of modifiable factors, and shared decision-making. It should not be used as a standalone numerical decision rule, to deny access to neuromodulation, or to replace multidisciplinary judgment. Prospective derivation, calibration, external validation, and decision-curve analysis are required before the STSS can be considered a clinical prediction rule.

## 1. Introduction

Spinal cord stimulation is an established treatment option for selected patients with refractory neuropathic pain, including persistent spinal pain syndrome type 2 (PSPS-T2), complex regional pain syndrome (CRPS), painful diabetic neuropathy (PDN), and other neuropathic pain states [1,2,3,4,5]. Contemporary consensus guidance emphasizes that SCS eligibility requires a structured, multidisciplinary assessment integrating clinical, psychosocial, technical, and patient-centered factors rather than relying on a single pain-intensity threshold [6].

Despite proven efficacy, SCS outcomes remain heterogeneous. Systematic reviews and long-term series demonstrate significant efficacy in appropriately selected patients; however, trial failure, loss of analgesia, explantation, complications, and incomplete functional recovery remain prevalent challenges [7,8,9]. This presents a clinical dilemma, as the literature contains numerous potentially relevant predictors that cannot be easily translated into a practical tool during routine consultation.

Economic considerations are also critical. Cost-effectiveness analyses generally support SCS in selected patients, particularly over longer time horizons; however unsuccessful trials and subsequent explantations increase costs without durable clinical benefit [10,11,12]. A structured pre-trial assessment protocol may support more consistent documentation, realistic expectation setting, and earlier optimization of modifiable factors. However, any such tool must avoid the methodological error of presenting an unvalidated score as a prediction rule.

Psychological and behavioral factors are particularly determinant. Systematic reviews have associated depression, anxiety, somatization, catastrophizing, low self-efficacy, and poor coping with poorer outcomes after lumbar surgery and SCS, although effect sizes vary across studies [13,14]. European and international selection frameworks similarly emphasize the need for integrated clinical and psychosocial assessment [15,16]. Computational and machine-learning approaches may eventually improve personalized prediction, but their routine clinical implementation use remains limited by requirements for the need for large datasets, infrastructure, external validation, and transparent reporting [17].

Equally important is how patients perceive their pain. A patient’s beliefs about pain controllability and meaning, together with the burden of fatigue and self-rated quality of life, shapes expectations, engagement with trialing, and the subjective appraisal of benefit. These experiential dimensions are frequently neglected when candidate assessment is reduced to diagnostic labels [18,19]. These self-perceptions are formed within an information environment that clinicians can no longer assume to be neutral. Exposure to health misinformation and disinformation can distort beliefs about the benefits and risks of neuromodulation, reinforce maladaptive illness narratives, and erode trust in clinical advice, thereby compromising health-related decision-making [20]. A structured pre-trial conversation should therefore seek to elicit, document, and, where necessary, recalibrate these perceptions, rather than treating psychological status as a static comorbidity.

Existing resources address related but distinct needs. Consensus guidelines [6,15] provide broad evidence-based recommendations but lack a compact, domain-by-domain bedside assessment structure. Machine-learning approaches remain constrained by infrastructure requirements, dataset size, and the absence of prospective external validation in routine clinical settings. Furthermore, no currently available tool combines multidomain clinical profiling with explicit methodological safeguards against score misuse. The Spinal Cord Stimulation Trial Success Score (STSS) is proposed to fill this specific gap.

The present article proposes the Spinal Cord Stimulation Trial Success Score (STSS) as an evidence-informed conceptual framework for structured candidate assessment. It is not presented as a validated prognostic model. This distinction is central: established methodological standards for prediction research require clear derivation methods, discrimination, calibration, external validation, and assessment of clinical utility before a tool can be used as a clinical prediction rule [21,22,23]. The STSS is therefore intended as a hypothesis-generating scaffold to support consistent documentation and shared decision-making pending prospective validation.

The role of the SCS screening trial itself has also become more nuanced. TRIAL-STIM found no superior six-month clinical outcome and no cost-effectiveness advantage for a screening-trial strategy compared with implantation without a separate trial in selected patients with neuropathic pain; its 36-month follow-up similarly found no long-term patient-outcome benefit from undertaking a screening trial [24,25]. These findings do not negate the need for careful candidacy assessment. Rather, they reinforce the rationale for a pre-procedural framework that documents indication, psychosocial readiness, modifiable factors, and patient goals without treating the temporary trial as the sole determinant of suitability.

## 2. Materials and Methods

### 2.1. Design and Scope

This work presents a structured narrative review and a conceptual framework proposal. It does not constitute a systematic review, meta-analysis, derivation study, or external validation study. The objective was to synthesize clinically accessible domains frequently reported in the SCS literature and to translate them into a transparent point-of-care tool for pre-trial assessment. Because the STSS was not derived from individual patient-level data, no estimates of discrimination, calibration, sensitivity, specificity, net benefit, or predictive values are reported. Consequently, the proposed point allocations should therefore be interpreted as a pragmatic semi-quantitative hierarchy based on consistency, clinical plausibility, and bedside feasibility rather than regression-derived weights.

### 2.2. Literature Source Identification

A targeted, structured narrative literature search was conducted to identify clinical trials, systematic reviews, registry studies, consensus guidelines, and selected methodological papers relevant to SCS candidate assessment, trial stimulation, and outcome interpretation. The search spanned PubMed/MEDLINE, Embase, the Cochrane Library, and Google Scholar, supplemented by manual screening of reference lists from key guidelines, systematic reviews, and landmark trials. The search covered publications available from database inception to May 2026. No formal language restrictions were applied, although the final synthesis prioritized peer-reviewed English-language articles and consensus recommendations with direct relevance to SCS practice (Table S1).

Because this was a narrative synthesis rather than a systematic review, the number of initially identified database records was not formally recorded and no PRISMA flow diagram was generated. Sources were selected for inclusion if they addressed at least one STSS domain, SCS trialing, patient selection, psychological assessment, opioid burden, smoking, body mass index, pain duration, health-economic implications, or prediction-model methodology. Sources were excluded if they focused primarily on technical reports without candidate-assessment relevance, abstracts without sufficient methodological detail, non-SCS neuromodulation studies, or articles not directly informing the conceptual framework.

### 2.3. Domain Selection

**Figure 1:**
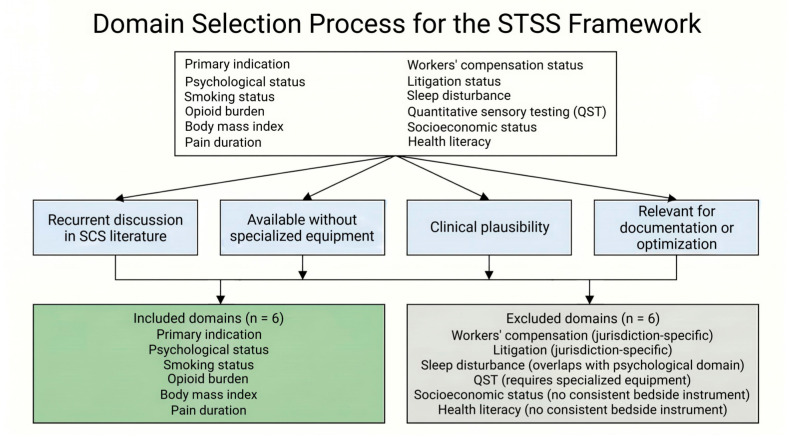
Domain selection process for the STSS framework. Candidate domains identified from the SCS literature were evaluated against four prespecified selection criteria: recurrent discussion in the SCS literature, availability during routine clinical consultation without specialized equipment, clinical plausibility, and potential relevance for documentation, optimization, or shared decision-making. Domains meeting all four criteria were included in the STSS (*n* = 6); domains failing one or more criteria were excluded (*n* = 6). Reasons for exclusion are indicated in parentheses. Abbreviations: QST, quantitative sensory testing; SCS, spinal cord stimulation; STSS, Spinal Cord Stimulation Trial Success Score.

Candidate domains were selected using four criteria: (1) recurrent discussion in more than one strand of the SCS literature; (2) availability during routine clinical consultation without specialized software or equipment; (3) clinical plausibility; and (4) potential relevance for documentation, optimization, or shared decision-making. Six domains met these criteria: primary indication, psychological status, smoking status, opioid burden, body mass index, and pain duration (Figure 1).

Several additional domains were considered but excluded. Workers’ compensation status and active litigation were omitted because their relevance is jurisdiction-specific and their operationalization varies substantially across healthcare systems, limiting cross-site applicability. Sleep disturbance was considered clinically plausible but insufficiently distinguished from the psychological domain for independent scoring at the bedside. Quantitative sensory testing (QST) was excluded because it requires specialized equipment and trained personnel not universally available in routine clinical consultation. Socioeconomic status and health literacy were recognized as contextually important but difficult to score consistently without validated, setting-specific instruments. These exclusions reflect the framework’s emphasis on bedside feasibility rather than a judgment that these factors are clinically unimportant.

### 2.4. Framework Construction and Methodological Safeguards

The STSS was intentionally framed as a clinical profile rather than a prediction rule. To reduce the risk of overinterpretation, the score categories use descriptive language rather than probability claims. The score is not intended to exclude patients from SCS, determine payer authorization, or replace multidisciplinary judgment. It is intended to make pre-trial assessment more explicit, reproducible, and transparent.

Beyond these tiers, the total score is deliberately additive and unweighted to keep the framework usable at the bedside. However, this approach introduces an explicit cost: an additive total assumes that domains contribute independently and approximately linearly, therefore failing to account for interactions between domains, nonlinear effects, or diagnosis-specific modifiers. The prognostic meaning of a given pain duration, for instance, may differ across PSPS-T2, CRPS, and painful diabetic neuropathy. These simplifications are defensible for a documentation aid but would be inadmissible in a prediction model, representing key issues that prospective derivation must resolve.

Future studies should evaluate whether the STSS satisfies accepted standards for prognostic model research, including TRIPOD-based reporting and risk-of-bias assessment using PROBAST or related tools [21,22,23]. Until such work is completed, the STSS should be solely a framework for structured reasoning.

### 2.5. Ethical Considerations

This article synthesizes previously published data and proposes a conceptual framework. No new patient-level data were collected; therefore, institutional review board approval was not required.

### 2.6. Quality Assessment

Because this is a narrative review and conceptual framework rather than a systematic review, no formal risk-of-bias assessment (e.g., QUADAS-2, ROBINS-I, or GRADE) was performed. The “evidence support” ratings reflect the authors’ qualitative synthesis of study design, consistency, and sample size across the selected literature; they are not formally graded ratings and should not be interpreted as standardized levels of evidence. Readers are referred to existing systematic reviews and meta-analyses cited herein for formal quality assessments.

## 3. Results

### 3.1. Evidence Basis for Candidate Domains

#### 3.1.1. Primary Indication

Primary pain diagnosis is the most clinically intuitive domain in SCS selection. Evidence robustly supports neuropathic leg-predominant pain after spine surgery, CRPS, and PDN, although outcome durability and functional benefit vary by clinical indication and neuromodulation modality [2,3,4,5,7,8,9]. Historical and current data also suggest that predominant axial pain, widespread pain, or poorly localized nociceptive pain may be less predictable than radicular or neuropathic pain patterns, although newer stimulation waveforms continue to expand therapeutic options [26,27,28].

For this reason, the STSS assigns the highest indication-related score to PSPS-T2 with predominant radicular pain and CRPS type I/II with a neuropathic limb pain phenotype, an intermediate score to PDN, and lower scores to mixed or axial-predominant presentations. CRPS type II is not excluded from the framework. Because clinical practice and several SCS evidence syntheses commonly discuss CRPS-related neuropathic limb pain as a broader indication, CRPS type II is grouped pragmatically with CRPS type I in Table 1. This grouping should not be interpreted as implying that the evidence base, sample size, pathophysiology, or generalizability are uniform across these conditions.

Similarly, assigning both PSPS-T2 with predominant radicular pain and CRPS to the highest indication tier is a pragmatic approximation rather than a claim of equal evidentiary strength. The evidence base for CRPS is more limited in sample size and generalizability than that for PSPS-T2; the shared tier reflects clinical plausibility and established indication status rather than statistical equivalence.

#### 3.1.2. Psychological Status

Psychological status was assigned substantial weight because SCS requires active engagement with trialing, programming, device use, expectation management, and behavioral adaptation. Reviews and cohort studies repeatedly associate depression, anxiety, somatization, catastrophizing, poor coping, low self-efficacy, and sleep interference with less favorable outcomes, although no single psychological factor should be considered absolute grounds for exclusion [13,14,29,30,31,32,33].

Recent narrative evidence suggests that psychological readiness may be mechanistically relevant rather than merely administrative, because maladaptive cognitive-affective profiles can interact with prefrontal-limbic regulation, reward processing, descending pain modulation, and central sensitization [34]. The STSS therefore treats psychological status as a central optimization domain. This should not be interpreted as a reason to exclude patients with treated or stable psychological comorbidity.

Operationalization of the psychological domain should not rely solely on unstructured clinical impressions. Where a pain psychologist is available, psychological domain scoring should preferably be informed by a formal pain psychology assessment. In settings without immediate access to specialized services, clinicians may use brief validated screening instruments to support reproducible scoring and identify patients requiring specialist referrals. Examples include the Patient Health Questionnaire-9 for depressive symptoms, the Generalized Anxiety Disorder-7 scale for anxiety, the Pain Catastrophizing Scale for catastrophizing, and the Pain Self-Efficacy Questionnaire for self-efficacy [35,36,37,38]. These instruments are not proposed as mandatory components of the STSS, but they represent the preferred operational pathway when formal psychological assessment is not immediately available to enhance consistency, reproducibility, and multidisciplinary communication. Clinicians using unstructured clinical impression alone should recognize that this reduces the comparability of STSS scores across settings, a limitation that future validation studies will need to address through standardized assessment protocols.

#### 3.1.3. Smoking Status

Current tobacco smoking has been associated with poorer pain outcomes and higher opioid use after SCS implantation in retrospective data [39]. Smoking may also increase general perioperative risk through impaired wound healing and infection-related mechanisms, although SCS-specific evidence remains less robust than evidence for major clinical indications or psychological screening. The STSS therefore treats active smoking as an unfavorable but potentially modifiable factor rather than as an absolute contraindication.

#### 3.1.4. Opioid Burden

The relationship between baseline opioid therapy and SCS outcome is clinically important, but the evidence is inconsistent. Claims-based and cohort data have associated greater preimplant opioid exposure with explantation or less favorable outcomes in some settings [40]. However, a large cohort of 467 permanently implanted patients found that preoperative opioid use and daily opioid dose were not associated with patient-reported improvement, pain-intensity reduction, or explantation risk [41]. Narrative synthesis also suggests that SCS may reduce opioid use in some populations, but causal interpretation remains limited by heterogeneity [42].

For these reasons, opioid burden is included as a contextual and modifiable clinical domain rather than as an exclusionary criterion. High-dose opioid therapy should prompt expectation setting, safety review, and consideration of supervised optimization, but current evidence does not support withholding otherwise well-indicated SCS solely because a patient uses opioids.

The opioid dose categories used in Table 1 (50 MME/day or less, 51 to 90 MME/day, and more than 90 MME/day) are pragmatic operational categories rather than validated SCS-specific cut-points. The more than 90 MME/day category reflects a commonly used high-dose opioid threshold in pain and public health literature, whereas the 50 MME/day boundary was used to distinguish lower-dose from intermediate-dose opioid exposure for bedside interpretability. These thresholds should be recalibrated in future derivation and validation studies.

#### 3.1.5. Body Mass Index

Obesity may influence SCS through technical, metabolic, inflammatory, and perioperative mechanisms. In a retrospective cohort of chronic spine-related pain patients, increasing BMI was negatively associated with SCS effectiveness at 6 and 12 months [43]. However, BMI is an imperfect proxy for adiposity, functional reserve, metabolic health, and technical complexity. The STSS therefore assigns only one point to BMI, recognizing its practical relevance while avoiding disproportionate weighting. The threshold of 30 kg/m^2^ follows a widely used clinical classification of obesity rather than a validated SCS-specific prognostic cut-point and should be recalibrated in future derivation studies.

#### 3.1.6. Pain Duration

Longer pain duration has been associated with less favorable SCS outcomes in long-term clinical series, possibly reflecting chronic central sensitization, behavioral adaptation to disability, deconditioning, and more complex psychosocial burden [44,45]. The evidence is not strong enough to justify rigid cutoffs. The 5-year threshold used in Table 1 was selected for operational simplicity and should not be interpreted as an evidence-derived or validated SCS-specific cut-point. In the STSS, pain duration is therefore included as a low-weight domain and should be interpreted in the broader clinical context.

### 3.2. The STSS Framework

**Figure 2:**
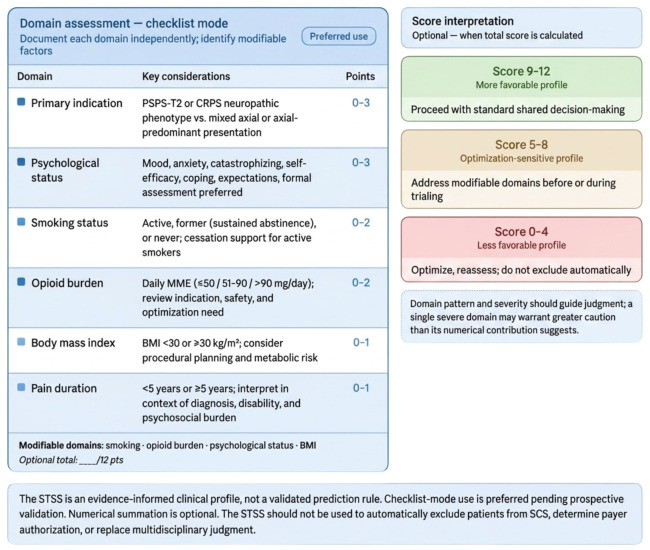
SCS Trial Success Score (STSS): domain checklist and descriptive categories. Left panel (preferred use): the six STSS domains presented as a structured checklist for independent assessment of each domain. Each row lists the domain name, key clinical considerations, and maximum point allocation. Color coding reflects domain weight: Primary indication and Psychological status (dark blue; 0–3 points each), Smoking status and Opioid burden (medium blue; 0–2 points each), and Body mass index and Pain duration (light blue; 0–1 point each). Modifiable domains (Smoking status, Opioid burden, Psychological status, and BMI) are listed in the panel footer; numerical summation to a total score (0–12 points) is presented as optional. Right panel (optional, when total score is calculated): three descriptive categories by total STSS score. Score 9–12 corresponds to a more favorable profile; Score 5–8 to an optimization-sensitive profile; Score 0–4 to a less favorable profile. A supplementary note in the right panel indicates that domain pattern and severity should guide clinical judgment alongside any calculated total. The disclaimer below the figure reflects the developmental status of the framework. Created in BioRender. Abbreviations: BMI, body mass index; CRPS, complex regional pain syndrome; MME, morphine milligram equivalents; PSPS-T2, persistent spinal pain syndrome type 2; SCS, spinal cord stimulation; STSS, Spinal Cord Stimulation Trial Success Score.

The proposed STSS consists of six domains that can be assessed during routine clinical evaluation. The total score ranges from 0 to 12 points (Figure 2, Table S3). The score is intended to summarize an evidence-aligned clinical profile, not to estimate an individual probability of SCS success. Until prospective validation is completed, the framework should preferentially be used as a structured checklist for documenting relevant domains, identifying modifiable factors, and supporting shared decision-making; numerical summation should remain optional.

### 3.3. Descriptive Categories and Clinical Use

The descriptive categories in Table 2 are intentionally non-probabilistic. The category boundaries were selected using a transparent post hoc rationale rather than statistical derivation. A score of 9 or above requires at least the two highest-weighted domains, primary indication and psychological status, to be rated favorably, reflecting the clinical centrality of these factors in the SCS literature. The midrange category (5 to 8) captures profiles in which one or more modifiable domains warrant attention but do not preclude trial consideration. The lower category (0 to 4) represents clustering of multiple unfavorable domains across the six dimensions. These boundaries should be regarded as provisional and subject to recalibration in future derivation studies. They should not be converted into treatment thresholds until prospective validation establishes calibration, discrimination, and clinical net benefit.

A total score can also conceal clinically important patterns and should not be read in isolation. A single severe red-flag domain, such as active and uncontrolled psychiatric instability, dominant secondary-gain concerns, or an unsafe opioid pattern requiring optimization, may warrant greater caution than its numerical contribution suggests and should not be offset by favorable scores in other domains. Conversely, a patient with several mild, modifiable concerns distributed across domains may be more readily prepared for trialing than a patient who reaches the same total through one severe domain. Clinicians should therefore weigh the severity and pattern of individual domains, not only their arithmetic sum; any domain rated at its lowest tier should prompt explicit discussion and targeted optimization regardless of the total score.

### 3.4. Evidence Map and Rationale for Weighting

Table 3 summarizes the evidentiary rationale and limitations for each domain. The table is designed to make the framework transparent and to help reviewers distinguish the STSS from a statistically derived model.

### 3.5. Intended and Non-Intended Use

Table 4 summarizes the intended and non-intended uses of the STSS. The framework is not intended to exclude patients from SCS, determine payer authorization, replace psychological assessment, or substitute for multidisciplinary judgment. Its intended uses include structured documentation of SCS candidacy domains, shared decision-making, identification of modifiable factors, communication within multidisciplinary teams, and hypothesis generation for future validation studies.

### 3.6. Scoring Uncertainty and Checklist Mode

Because the STSS point values and category thresholds have not been statistically derived, summation of the total score should be considered optional. Pending prospective validation, checklist-mode use is the preferred interim application. In this mode, each domain is documented separately, modifiable factors are identified, and the framework supports transparent shared decision-making without implying that the total score is a validated numerical decision rule.

This distinction is important for publishing the framework in the absence of an original validation cohort. The manuscript proposes an explicit developmental stage: domain identification and transparent framework construction. It does not claim that the current point system is calibrated, externally validated, or ready for threshold-based clinical decision-making.

### 3.7. Relationship to Existing Selection Approaches

Table 5 places the STSS in context. The framework is not intended to replace consensus recommendations, psychological instruments such as the Psychological Evaluation Tool for Spinal Cord Stimulation Candidacy (PETSCSC), integrated multidisciplinary assessment, or future machine-learning models. Its intended niche is narrower: to provide a concise bedside structure for documenting key domains before a trial or implant decision. More detailed assessment of psychological domains; PETSCSC correlated with selected postoperative patient-reported outcomes in a small prospective cohort [46]. Unlike the STSS, however, PETSCSC focuses exclusively on psychological assessment and does not integrate clinical indication, opioid burden, smoking status, body mass index, or pain duration into a multidomain clinical profile.

### 3.8. Modifiable Domains

Smoking: Patients who actively smoke should receive counseling and evidence-based cessation support. Smoking should be discussed as a modifiable perioperative and outcome-related risk factor rather than as an absolute contraindication.

Opioid burden: High-dose opioid therapy should prompt review of indication, safety, function, and expectations. Gradual dose optimization may be reasonable when clinically appropriate, but current evidence does not justify denying SCS solely on the basis of preoperative opioid use.

Psychological status: Untreated depression, anxiety, marked catastrophizing, unrealistic expectations, and poor coping should trigger psychological assessment and targeted intervention. Cognitive-behavioral therapy, acceptance-based approaches, pain education, expectation management, and behavioral activation may improve readiness for trialing.

Body mass index and general health: Weight-related counseling should be individualized. In patients with morbid obesity or major metabolic comorbidity, procedural planning and perioperative risk mitigation may be more relevant than short-term weight loss alone.

### 3.9. Clinical Application Examples

The following examples illustrate how the STSS may structure discussion; they are not intended to estimate a validated probability of success.

Example 1: More favorable profile. A 52-year-old patient has PSPS-T2 with predominant L5 radicular pain (3 points), no significant psychological risk factors (3 points), former smoking with sustained abstinence (1 point), opioid use of 40 MME/day (2 points), BMI 28 kg/m^2^ (1 point), and pain duration of 3 years (1 point). Total STSS: 11. This profile supports standard shared decision-making and trial planning.

Example 2: Optimization-sensitive profile. A 58-year-old patient has PSPS-T2 with mixed axial and radicular pain (1 point), treated depression with stable symptoms (2 points), never smoked (2 points), opioid use of 80 MME/day (1 point), BMI 32 kg/m^2^ (0 points), and pain duration of 6 years (0 points). Total STSS: 6. This profile supports trial consideration with explicit optimization of expectations, opioid safety, functional goals, and weight-related procedural planning.

Example 3: Less favorable profile. A 48-year-old patient has axial-predominant low back pain without clear neuropathic phenotype (0 points), untreated depression and high catastrophizing (1 point), active smoking (0 points), opioid use of 120 MME/day (0 points), BMI 34 kg/m^2^ (0 points), and pain duration of 12 years (0 points). Total STSS: 1. This profile should prompt optimization and reassessment rather than automatic exclusion. Psychological care, smoking cessation support, opioid-safety review, and discussion of alternative therapies should precede final trial planning. Management should preferably occur within a multidisciplinary pathway involving the implanting physician or neurosurgeon, a pain physician, a dedicated pain psychologist, and, when appropriate, physiotherapy and addiction or opioid-safety support.

### 3.10. Suggested Implementation Pathway

To clarify how the STSS is intended to function in practice, and to distinguish it further from a prediction model, the framework is best embedded in the existing multidisciplinary pathway as a sequence of documentation and optimization steps rather than as a gate that admits or excludes candidates.

At the initial consultation, each of the six domains is documented explicitly, establishing a transparent baseline and a shared vocabulary for the team. Domains rated below their maximum are read as a list of potentially modifiable targets: psychological concerns prompt referral for formal pain-psychology assessment and, where indicated, cognitive-behavioral or acceptance-based intervention; high-dose or unsafe opioid exposure prompts a safety and indication review with consideration of supervised optimization; active smoking prompts cessation support; and elevated body mass index prompts individualized counseling and perioperative risk planning. The same documentation then informs procedural planning and the shared decision-making conversation, in which the identified domains, the strengths and limits of the supporting evidence, and the patient’s own goals and expectations are discussed openly. Candidacy is reassessed once modifiable factors have been addressed, so that a low initial profile leads to optimization and review rather than to exclusion. Used in this way, the STSS supports the multidisciplinary process by making its reasoning explicit and reproducible; it does not replace clinical judgment, psychological assessment, or the team’s final decision.

## 4. Discussion

The STSS is proposed as a pragmatic, evidence-informed framework for organizing SCS candidate assessment. Its main value lies in fostering clinical transparency rather than prognostic accuracy, making explicit which clinical domains were considered, which modifiable factors were identified, and how uncertainty was communicated to the patient.

A further safeguard involves distinguishing trial response from long-term implanted SCS durability. Emerging data, including the TRIAL-STIM, question whether a separate screening trial provides incremental clinical or economic benefit in selected populations, whereas consensus guidance still recommends trialing in most non-cancer pain indications [6,24,25]. The STSS should therefore be read as a framework for pre-procedural candidacy assessment rather than as an argument for mandatory trial stimulation or as a predictor of long-term explantation risk.

The Taylor meta-regression is particularly relevant to the STSS because it demonstrates both the promise and the difficulty of prognostication in SCS: across 74 studies and 3025 patients, pain relief was heterogeneous, but multivariable meta-regression did not identify stable patient- or technology-level predictors at the study level [47]. This supports the cautious framing adopted here. The STSS should organize clinical reasoning; it should not be presented as if the literature already provides reliable patient-level prediction. In this context, the STSS responds to these challenges by shifting emphasis away from predicting technical trial success alone toward multidimensional pre-neuromodulation patient profiling, standardized documentation, and identification of modifiable domains.

The STSS does not compete with existing approaches. Its contribution is narrower and more specific: to make pre-trial assessment explicit, reproducible, and transparent at the point of clinical consultation, without requiring specialized software, extensive psychological training, or large datasets. This positions the STSS as a documentation and communication tool rather than a prediction instrument, a distinction that defines both its intended use and its current limitations.

The most important methodological safeguard is the distinction between a conceptual framework and a clinical prediction model. Prediction models require derivation from appropriate patient-level data, internal validation, calibration assessment, external validation, and evaluation of clinical utility. The STSS has not yet undergone these steps; therefore, its categories cannot be interpreted as validated probabilities of trial success [21,22,23].

This distinction is central to the methodological defensibility of the framework, because it clarifies that the STSS is intended to support structured clinical profiling rather than validated individual outcome prediction. Without validation, a score-based tool may be criticized for unjustified weighting, arbitrary thresholds, and potential misuse as an exclusionary instrument. By presenting the STSS as a structured profile, adding an evidence map, softening categorical language, and explicitly prohibiting its use as a stand-alone eligibility rule, the article becomes methodologically more defensible.

Primary indication and psychological status receive the highest point allocations because they are clinically central and repeatedly emphasized in the SCS literature. However, these weights should be considered provisional. The same applies to the lower weights assigned to smoking, opioid burden, BMI, and pain duration. The purpose of weighting is bedside usability, not statistical precision.

The additive structure of the STSS does not model interactions between domains. In clinical practice, the clustering of unfavorable factors such as untreated depression, high catastrophizing, active smoking, and high-dose opioid exposure may carry greater clinical concern than the arithmetic sum of individual domain scores suggests. Clinicians should therefore interpret the total score, when calculated, in conjunction with the pattern and clustering of individual domains.

The opioid domain illustrates why caution is essential. Some datasets suggest that high-dose opioid exposure may be associated with less favorable outcomes or explantation, whereas other large cohort data do not support preoperative opioid use as an independent predictor. The STSS therefore treats opioid burden as a modifiable context variable rather than as a reason to withhold SCS from otherwise appropriate candidates [40,41,42].

The psychological domain also requires careful interpretation. Psychological comorbidity is common in chronic pain and should not be used to stigmatize or exclude patients reflexively. The relevant question is whether psychological factors are untreated, unstable, severe, or likely to impair engagement with the trial and long-term therapy. Treated and stable mood or anxiety disorders can be compatible with SCS candidacy when expectations and support systems are appropriate [13,14,29,30,31,32,33,34].

The STSS also complements, rather than competes with, existing multidisciplinary and electronic assessment approaches. Integrated assessment frameworks emphasize that SCS selection should incorporate clinical phenotype, psychosocial factors, patient goals, and expected functional benefit [15,16]. Machine-learning approaches may eventually refine prediction, but they currently require robust datasets, careful validation, and transparent reporting before routine clinical use [17,21,22,23].

The STSS also differs from the PETSCSC in scope and intended use. The PETSCSC is a psychology-focused instrument designed to structure preoperative psychological evaluation and has shown preliminary correlations with selected SCS outcomes in a small prospective cohort [46]. In contrast, the STSS is not a psychological screening instrument; it integrates psychological status with indication, smoking, opioid burden, BMI, and pain duration to support multidomain pre-trial documentation. The two approaches should therefore be viewed as complementary rather than competing.

Ethically, the STSS should be used to support access through better preparation, not to restrict access through simplistic scoring. A low score should trigger discussion, optimization, and reassessment. A high score should not guarantee success or reduce the need for careful informed consent. Used in this limited way, the STSS may provide a practical bridge between broad consensus recommendations and the realities of bedside consultation.

## 5. Limitations

This article has several limitations. First, it is a narrative synthesis rather than a systematic review with a reproducible PRISMA flow diagram. The evidence map improves transparency but does not eliminate selection bias. Second, the point values are not statistically derived and should be considered provisional. Third, outcome definitions in SCS vary widely across studies, including trial success, pain reduction, functional improvement, patient satisfaction, explantation, opioid reduction, and quality of life. Additionally, the psychological domain may be operationalized inconsistently across clinical settings, ranging from formal psychometric assessment to unstructured clinical impression, which limits the reproducibility and cross-site comparability of STSS scores pending standardization of assessment protocols. The instrument-to-tier anchors provided in Supplementary Table S2 are also illustrative and have not been empirically derived; the score ranges mapped to each psychological scoring tier have not been validated against SCS outcomes, and the appropriateness of standard cut-points from general chronic pain populations may not generalize to SCS candidate populations. Fourth, the STSS may not perform equally across conventional, high-frequency, burst, closed-loop, or other stimulation paradigms. Fifth, several candidate domains, including workers’ compensation status, litigation, socioeconomic status, sleep disturbance, and quantitative sensory testing, were considered but excluded on the basis of the criteria described in Section 2.3; their exclusion reflects pragmatic constraints rather than a judgment about clinical importance.

Most importantly, the STSS has not been validated in a derivation or external validation cohort. It should therefore remain a hypothesis-generating framework until prospective validation is completed. Finally, the STSS domains, point allocations, and tier boundaries have not undergone formal content or face validity assessment; a structured expert consensus process, such as a Delphi or modified Delphi procedure, would be required to establish content validity indices before the framework is used in prospective research.

## 6. Future Directions

Before patient-level validation, an efficient intermediate step would be a formal assessment of expert face and content validity. A small Delphi or modified Delphi panel should ideally include implanting physicians or neurosurgeons, pain physicians, pain psychologists, physiotherapists, methodologists, and, where feasible, patient representatives. Panel members could rate each STSS domain for relevance, clarity, feasibility, risk of misuse, and need for rewording. Reporting item-level and scale-level content validity indices would not validate predictive performance, but it would strengthen the framework by demonstrating expert agreement on content and usability before registry-based derivation.

Prospective validation should proceed in phases. First, consecutive SCS trial candidates should be enrolled in a multicenter registry, with all six STSS domains recorded before trialing by assessors blinded to outcome, where feasible. Second, candidate weights and thresholds should be refined using prespecified statistical methods. Third, the refined model should undergo external validation in an independent cohort. Discrimination should be reported using the area under the receiver operating characteristic curve, but calibration, decision curve analysis, and clinical net benefit are equally important. Fourth, subgroup performance should be examined by indication, stimulation paradigm, trial design, and psychological profile.

Core registry variables should include all STSS domains, detailed pain phenotype, psychological instruments, stimulation modality, trial type, programming strategy, functional goals, adverse events, explantation, opioid trajectory, and patient-reported global impression of change.

If prospective validation demonstrates acceptable calibration, discrimination, and net benefit, the STSS could be refined into a formal clinical prediction model. Until then, its role should remain limited to structured assessment and shared decision-making.

## 7. Conclusions

The STSS provides an evidence-informed conceptual framework for organizing pre-trial assessment in spinal cord stimulation. It summarizes six clinically accessible domains: primary indication, psychological status, smoking status, opioid burden, body mass index, and pain duration. The framework may help clinicians document candidacy, identify modifiable factors, and structure shared decision-making.

The STSS is not a validated clinical prediction rule and should not be used to deny access to neuromodulation. Prospective derivation, calibration, external validation, and decision curve analysis are required before probability-based interpretation or threshold-based decision-making can be justified.

## Figures and Tables

**Table 1:** SCS Trial Success Score (STSS) components.

Domain	Criteria	Points
Primary indication	PSPS-T2 with predominant radicular pain ^†^	3
	CRPS type I/II with neuropathic limb pain phenotype ^†^	3
	Painful diabetic neuropathy	2
	PSPS-T2 with mixed axial and radicular pain	1
	Axial-predominant pain, widespread pain, or other less evidence-aligned indication	0
Psychological status	No clinically significant psychological risk factors identified	3
	Treated and stable mood or anxiety disorder; realistic expectations	2
	Untreated depression, anxiety, marked catastrophizing, low self-efficacy, or poor coping	1
	Active somatization, severe uncontrolled psychiatric instability, or dominant secondary-gain concerns	0
Smoking status	Never smoker	2
	Former smoker or sustained abstinence	1
	Active smoker	0
Opioid burden	No opioids or low-dose opioids (≤50 MME/day)	2
	Moderate-dose opioids (51–90 MME/day)	1
	High-dose opioids (>90 MME/day) or unsafe opioid pattern requiring optimization	0
Body mass index	BMI < 30 kg/m^2^	1
	BMI ≥ 30 kg/m^2^	0
Pain duration	<5 years	1
	≥5 years	0

^†^ Equal point allocation for PSPS-T2 and CRPS does not imply equivalent evidence strength, effect size, or generalizability. The evidence base for CRPS is more limited in sample size and generalizability than that for PSPS-T2; the shared tier reflects clinical plausibility and established indication status rather than statistical equivalence. Abbreviations: BMI, body mass index; CRPS, complex regional pain syndrome; MME, morphine milligram equivalents; PSPS-T2, persistent spinal pain syndrome type 2; SCS, spinal cord stimulation; STSS, Spinal Cord Stimulation Trial Success Score.

**Table 2:** STSS descriptive categories and suggested clinical use.

Score Range	Descriptive Category ^†^	Interpretation	Suggested Clinical Use
9–12	More favorable profile	Multiple domains align with factors commonly associated with more favorable SCS outcomes.	Proceed with standard shared decision-making, documentation of expectations, and trial planning.
5–8	Optimization-sensitive profile	One or more modifiable domains may warrant attention before or during trialing.	Consider psychological, opioid-related, smoking-related, weight-related, or expectation-focused optimization; proceed when clinically justified.
0–4	Less favorable profile	Several domains raise concern for reduced engagement, response, durability, or procedural risk.	Do not exclude automatically. Discuss uncertainty, optimize modifiable factors, consider alternative therapies, and reassess candidacy in a multidisciplinary setting.

^†^ These categories are illustrative communication labels only. No evidence supports their use as clinical decision thresholds, eligibility criteria, or payer authorization tools. The descriptive labels (more favorable, optimization-sensitive, less favorable) reflect discussion-starting profiles, not validated prognostic strata.

**Table 3:** Evidence map for STSS domains.

STSS Domain	Evidence Basis	Direction of Association	Approximate Evidentiary Strength	Key Caveat
Primary indication	RCTs, systematic reviews, long-term series, and consensus recommendations [2,3,4,5,6,7,8,9,26,27,28,29]	More favorable profiles generally involve neuropathic, radicular, CRPS, or PDN presentations.	Moderate to high, depending on indication	Evidence varies by waveform, lead type, outcome definition, and pain phenotype.
Psychological status	Systematic reviews, prospective and retrospective cohorts, qualitative studies, and mechanistic narrative synthesis [13,14,29,30,31,32,33,34]	Untreated depression, anxiety, catastrophizing, poor coping, low self-efficacy, and somatization are generally unfavorable.	Moderate	No single psychological construct is determinative; treated/stable conditions should not preclude SCS.
Smoking status	Retrospective SCS cohort data and broader surgical risk literature [39]	Active smoking is generally unfavorable.	Low to moderate	Smoking should be framed as modifiable risk rather than automatic exclusion.
Opioid burden	Claims analyses, cohort studies, and narrative synthesis [40,41,42]	High-dose opioids may be unfavorable in some datasets, but findings are inconsistent.	Mixed	A large cohort found no association between preoperative opioid use or dose and SCS outcome.
Body mass index	Retrospective cohort data [43]	Higher BMI may be associated with reduced effectiveness and technical complexity.	Low to moderate	BMI is an imperfect proxy for procedural difficulty and metabolic risk.
Pain duration	Long-term clinical series [44,45]	Longer pain duration may be unfavorable.	Low to moderate	Cutoffs are not validated; duration interacts with diagnosis, disability, and psychosocial factors.

Note: “Evidence support within SCS literature” is a qualitative descriptor reflecting the consistency, volume, and design of the available studies for each domain. It is an informal summary intended to help the reader gauge relative confidence; it is not a formal graded rating such as GRADE, and the labels (low, low to moderate, moderate, moderate to high, mixed) should not be interpreted as standardized levels of evidence. Abbreviations: BMI, body mass index; CRPS, complex regional pain syndrome; PDN, painful diabetic neuropathy; RCTs, randomized controlled trials; SCS, spinal cord stimulation; STSS, Spinal Cord Stimulation Trial Success Score.

**Table 4:** Intended and non-intended use of the STSS.

Intended Use	Non-Intended Use
Structured documentation of SCS candidacy domains; shared decision-making; identification of modifiable factors; communication within multidisciplinary teams; hypothesis generation for future validation studies.	Automated exclusion from SCS; payer denial; replacement of psychological assessment; replacement of multidisciplinary judgment; estimation of an individualized probability of trial success; use as a validated clinical prediction rule before validation.

**Table 5:** Relationship of the STSS to existing SCS selection and assessment approaches.

Approach	Main Contribution	Main Limitation	Relationship to STSS
ASRA/NANS/ESRA consensus guidance	Evidence-based recommendations on patient selection, psychosocial screening, and trial conduct [6].	Broad guidance rather than a compact bedside scoring structure.	STSS operationalizes selected domains into a concise documentation framework.
Integrated European assessment frameworks	Multidomain assessment of clinical and psychosocial factors [15].	May require structured processes or tools not universally available in routine clinics.	STSS offers a simplified, transparent summary for bedside use.
Psychological screening tools, including PETSCSC	More detailed assessment of psychological domains; PETSCSC correlated with selected postoperative patient-reported outcomes in a small prospective cohort [46].	Domain-specific; small cohorts; not a comprehensive clinical profile.	STSS incorporates psychology as one high-weight domain but does not replace formal psychological assessment.
Meta-regression and systematic predictor reviews	Demonstrate heterogeneity and the difficulty of identifying stable predictors across studies [47].	Study-level analyses may not provide patient-level prediction.	STSS acknowledges uncertainty and uses domains as a framework rather than a validated prediction model.
Machine-learning approaches	Potential for individualized prediction with larger datasets [17].	Require infrastructure, transparent reporting, external validation, and clinical utility assessment.	STSS prioritizes interpretability and immediate clinical usability.
TRIAL-STIM evidence on screening trials	Questions whether a separate screening trial improves outcomes or cost-effectiveness [24,25].	Does not eliminate the need for structured candidacy assessment.	STSS focuses on pre-procedural profiling and shared decision-making, not on defending mandatory trialing.

Abbreviations: ASRA, American Society of Regional Anesthesia and Pain Medicine; ESRA, European Society of Regional Anaesthesia and Pain Therapy; PETSCSC, Psychological Evaluation Tool for Spinal Cord Stimulation Candidacy; SCS, spinal cord stimulation; STSS, Spinal Cord Stimulation Trial Success Score; TRIAL-STIM, Trial Screening for Spinal Cord Stimulation Study.

## Data Availability

No new data were created or analyzed in this study.

## Supplementary Materials

The following supporting information can be downloaded at https://www.mdpi.com/article/10.3390/jcm15134849/s1. Table S1: literature search strategy, including database-specific search strings, the date the searches were run, the staged screening procedure, inclusion and exclusion criteria, the principal sources informing each STSS domain, and the rationale for including or excluding each candidate domain; Table S2: illustrative operational anchors mapping the PHQ-9, GAD-7, Pain Catastrophizing Scale, and Pain Self-Efficacy Questionnaire onto the four psychological scoring tiers; Table S3: illustrative numerical prototype showing the domain criteria with indicative point allocations (0–12 total) for use as a research scaffold in future derivation studies only; numerical summation is not required for clinical use of the framework.

## Abbreviations

The following abbreviations are used in this manuscript:

ASRA	American Society of Regional Anesthesia and Pain Medicine
BMI	Body mass index
CRPS	Complex regional pain syndrome
ESRA	European Society of Regional Anaesthesia and Pain Therapy
MME	Morphine milligram equivalents
NANS	North American Neuromodulation Society
PDN	Painful diabetic neuropathy
PETSCSC	Psychological Evaluation Tool for Spinal Cord Stimulation Candidacy
PROBAST	Prediction model Risk Of Bias ASsessment Tool
PSPS-T2	Persistent spinal pain syndrome type 2
QST	Quantitative sensory testing
RCT	Randomized controlled trial
SCS	Spinal cord stimulation
STSS	Spinal Cord Stimulation Trial Success Score
TRIAL-STIM	Trial Screening for Spinal Cord Stimulation Study
TRIPOD	Transparent Reporting of a Multivariable Prediction Model for Individual Prognosis or Diagnosis
