# Supplementary material for: The Spinal Cord Stimulation Trial Success Score (STSS): A Narrative Review and Evidence-Informed Conceptual Framework for Structured Candidate Assessment
Source: J Clin Med. 2026 Jun 23;15(13):4849. doi: 10.3390/jcm15134849 (PMC13360793; doi:10.3390/jcm15134849)
Supplement: Supplementary file 1 [file jcm-15-04849-s001.zip › jcm-4355223-supplementary.pdf]

Supplementary Materials

"The Spinal Cord Stimulation Trial Success Score (STSS): A Narrative Review and Evidence-Informed Conceptual Framework for Structured Candidate Assessment"

Journal of Clinical Medicine

Table S1. Literature Search Strategy and Domain Inclusion/Exclusion Rationale

Section A: Database search strings and parameters

Database	Search string (Boolean)	Date searched	Notes
**PubMed / MEDLINE**	("spinal cord stimulation"[MeSH] OR "spinal cord stimulation"[tiab] OR "neuromodulation"[tiab]) AND ("patient selection"[tiab] OR "candidate assessment"[tiab] OR "trial stimulation"[tiab] OR "outcome predict*"[tiab] OR "predictors"[tiab]) AND ("chronic pain"[MeSH] OR "neuropathic pain"[MeSH] OR "failed back surgery"[tiab] OR "CRPS"[tiab] OR "diabetic neuropath*"[tiab] OR "persistent spinal pain"[tiab])	May 2026	Supplemented by domain-specific sub-searches; reference lists of retrieved guidelines and systematic reviews also screened.
**Embase**	('spinal cord stimulation'/exp OR 'spinal cord stimulation':ti,ab) AND ('patient selection'/exp OR 'candidate selection':ti,ab OR 'trial stimulation':ti,ab OR 'outcome prediction':ti,ab) AND ('chronic pain'/exp OR 'neuropathic pain'/exp OR 'failed back surgery syndrome':ti,ab OR 'CRPS':ti,ab OR 'diabetic neuropathy'/exp OR 'persistent spinal pain syndrome':ti,ab)	May 2026	English language filter applied. Animal studies excluded.
**Cochrane Library**	"spinal cord stimulation" AND ("patient selection" OR "candidate" OR "predictors" OR "psychological" OR "opioid") — Title, Abstract, Keywords	May 2026	Cochrane Reviews and Protocols; CENTRAL searched separately for RCTs.
**Google Scholar**	"spinal cord stimulation" "patient selection" — iterative searches with domain-specific terms listed below	May 2026	First 20 pages screened per query. Used for grey literature, guidelines, and recent publications.

Domain-specific supplementary search strings (PubMed syntax; equivalent terms applied in all databases):

Domain	Search string
**Psychological status**	("spinal cord stimulation") AND ("depression" OR "anxiety" OR "catastrophiz*" OR "psychological assessment" OR "psychosocial" OR "coping" OR "self-efficacy" OR "somatization")
**Smoking status**	("spinal cord stimulation") AND ("smoking" OR "tobacco" OR "cigarette" OR "nicotine" OR "smoking cessation")
**Opioid burden**	("spinal cord stimulation") AND ("opioid" OR "morphine milligram equivalent" OR "MME" OR "opioid dose" OR "opioid use")
**Body mass index**	("spinal cord stimulation") AND ("obesity" OR "body mass index" OR "BMI" OR "overweight")
**Pain duration**	("spinal cord stimulation") AND ("pain duration" OR "chronicity" OR "duration of pain" OR "central sensitization")
**Primary indication**	("spinal cord stimulation") AND ("patient selection" OR "indication" OR "PSPS" OR "CRPS" OR "diabetic neuropathy" OR "persistent spinal pain syndrome")
**Methodological / prediction models**	("spinal cord stimulation") AND ("prediction model" OR "TRIPOD" OR "PROBAST" OR "calibration" OR "clinical utility")
**Self-perception, fatigue, QoL**	("spinal cord stimulation") AND ("fatigue" OR "quality of life" OR "self-efficacy" OR "self-perception" OR "HRQoL")
**Patient beliefs / misinformation**	("spinal cord stimulation") AND ("misinformation" OR "patient expectations" OR "shared decision" OR "health literacy")

Screening procedure: *Records were assessed by the first author against the inclusion and exclusion criteria described in Section 2.2 of the main manuscript. Because this was a narrative synthesis, a formal PRISMA flow diagram and inter-rater agreement assessment were not generated. Principal sources informing each retained domain are listed in Section B below.*

Section B: Domain inclusion/exclusion rationale and principal sources

Domain	Decision	Key supporting sources (examples)	Rationale
**Primary indication**	Included	Kumar et al. 2007 [2]; North et al. 2005 [3]; Kemler et al. 2000 [4]; Petersen et al.	Recurrent in guidelines and landmark trials; evidence for established SCS indications; directly relevant to candidacy assessment.

Domain	Decision	Key supporting sources (examples)	Rationale
		2021 [5]; Shanthanna et al. 2023 [6]; Turner et al. 2004 [8]	
**Psychological status**	Included	Celestin et al. 2009 [13]; Sparkes et al. 2010 [14]; Thomson et al. 2022 [15]; Doleys 2006 [30]; Marcinkowska et al. 2026 [34]	Repeated association with SCS outcomes across study designs; assessable with validated brief instruments; central to multidisciplinary pre-trial assessment.
**Smoking status**	Included	Mekhail et al. 2018 [39]	SCS-specific cohort data; easily ascertained; clinically plausible mechanism; modifiable pre-trial.
**Opioid burden**	Included	Sharan et al. 2018 [40]; Poulsen et al. 2022 [41]; Rupp et al. 2022 [42]	Frequently discussed in SCS outcomes literature; relevant for expectation setting and safety review.
**Body mass index**	Included	Mekhail et al. 2019 [43]	SCS-specific cohort data; routinely documented; low weight reflects limited and indirect evidence.
**Pain duration**	Included	Kumar et al. 2006 [44]; Kumar et al. 1991 [45]	Discussed in long-term SCS series; easily ascertained; biological plausibility; low weight reflects uncertain threshold.
**Workers' compensation / Litigation**	Excluded	N/A	Jurisdiction-specific; operationalisation varies across healthcare systems; inclusion would limit cross-site applicability.
**Sleep disturbance**	Excluded	N/A	Plausible but insufficiently distinct from psychological domain; overlap with depression/medication makes isolated scoring unreliable.
**QST**	Excluded	N/A	Requires specialised equipment and trained personnel not available at routine consultation.
**Socioeconomic status**	Excluded	N/A	Not scorable consistently without validated, setting-specific instruments at routine consultation.
**Health literacy**	Excluded	N/A	Same rationale as socioeconomic status; not operationalisable consistently at bedside.

Table S2. Illustrative Operational Anchors for the Psychological Domain of the STSS

These anchors are illustrative worked examples to support reproducible documentation of the psychological domain. They are NOT validated SCS-specific diagnostic thresholds. Clinical judgement of treatment status, stability, and expected engagement takes precedence over any single instrument score. Instrument cut-offs cited are general-population or chronic-pain-population norms and have not been calibrated against SCS outcomes.

Tier	Descriptor	PHQ-9	GAD-7	PCS	PSEQ
**3**	No significant psychological risk factors	PHQ-9 < 5 OR clinician documents absence of significant depressive symptoms	GAD-7 < 5 OR clinician documents absence of significant anxiety; expectations assessed as realistic	PCS ≤ 20; no marked helplessness, rumination, or magnification on clinical interview	PSEQ > 40; patient expresses confidence in managing daily activities despite pain
**2**	Treated and stable mood or anxiety disorder; realistic expectations	PHQ-9 5–14 AND condition currently treated, stable ≥ 3 months	GAD-7 5–14 AND condition currently treated; SCS expectations assessed as realistic and grounded	PCS 21–29; mild catastrophizing present but not dominant; patient engaged with pain management	PSEQ 20–40 (moderate); patient has functional goals despite pain concerns
**1**	Untreated depression/anxiety, marked catastrophizing, or poor coping	PHQ-9 ≥ 10 AND untreated OR PHQ-9 ≥ 15 even if recently commenced treatment	GAD-7 ≥ 10 AND untreated OR anxiety-driven pain beliefs poorly calibrated	PCS ≥ 30 (clinically significant); marked helplessness, rumination, or magnification; OR avoidant/passive coping on interview	PSEQ < 20 (low); outcome expectations unrealistically high or low; little confidence in self-management
**0**	Active somatization, severe uncontrolled psychiatric instability, or dominant secondary-gain concerns	PHQ-9 ≥ 15 untreated OR active suicidal ideation OR comorbid severe untreated psychiatric condition	Severe anxiety disorder (GAD-7 ≥ 15) untreated; OR health anxiety with persistent catastrophic illness belief unmodified by reassessment; OR clearly unrealistic expectations not modifiable on discussion	Extreme catastrophizing (PCS ≥ 38) with denial of any psychosocial contribution; major somatization pattern; ALL suffering attributed to untreatable organic pathology	PSEQ < 10 or explicit denial of any agency; motivation for SCS appears primarily medicolegal or compensation-driven; dominant secondary gain clearly influencing consultation

PHQ-9 [35]; GAD-7 [36]; Pain Catastrophizing Scale [37]; Pain Self-Efficacy Questionnaire [38] (reference numbers as in main manuscript). Cut-offs are illustrative anchors requiring local standardisation; they have not been derived from SCS-specific validation studies.

Table S3. Illustrative Numerical Prototype — Research Scaffold Only

This table presents the indicative point allocations (0–12 total) for use as a research scaffold in future derivation studies. Numerical summation is NOT required for routine clinical use of the STSS. The preferred clinical application is domain-by-domain documentation using the checklist in Table 1 of the main manuscript. These point allocations have not been statistically derived, calibrated, or externally validated. They should not be interpreted as probabilities of trial success, used as eligibility thresholds, or applied to determine payer authorisation. The equal point allocation for PSPS-T2 and CRPS does not imply equivalent evidence strength, effect size, or durability of benefit.

Domain	Criteria	Points
**Primary indication**	PSPS-T2 with predominant radicular pain	**3**
	CRPS type I/II with neuropathic limb pain phenotype	**3**
	Painful diabetic neuropathy	**2**
	PSPS-T2 with mixed axial and radicular pain	**1**
	Axial-predominant, widespread, or other less evidence-aligned indication	**0**
**Psychological status**	No clinically significant psychological risk factors	**3**
	Treated and stable mood or anxiety disorder; realistic expectations	**2**
	Untreated depression/anxiety, marked catastrophizing, low self-efficacy, or poor coping	**1**
	Active somatization, severe uncontrolled psychiatric instability, or dominant secondary-gain concerns	**0**
**Smoking status**	Never smoker	**2**
	Former smoker or sustained abstinence	**1**
	Active smoker	**0**
**Opioid burden**	None or ≤50 MME/day	**2**
	51–90 MME/day	**1**
	>90 MME/day or unsafe opioid pattern requiring optimisation	**0**
**Body mass index**	BMI <30 kg/m²	**1**
	BMI ≥30 kg/m²	**0**
**Pain duration**	<5 years	**1**
	≥5 years	**0**

Descriptive categories (optional, provisional): Score 9–12 = more favorable profile; Score 5–8 = optimization-sensitive profile; Score 0–4 = less favorable profile. These labels are communication categories, not validated risk strata. A single severe red-flag domain should not be offset by favourable scores in other domains.
